# Prognostic Value of Maximal and Mean Lactate-to-Albumin (LAR), C-Reactive Protein-to-Albumin (CAR), and Procalcitonin-to-Albumin (PAR) Ratios Beyond ICU Admission in Critically Ill Patients

**DOI:** 10.3390/jcm15124698

**Published:** 2026-06-17

**Authors:** Krzysztof Żerdziński, Michał Gałuszewski, Julita Janiec, Michał Skrzypek, Łukasz J. Krzych

**Affiliations:** 1 Students Department “#Intensywna_Po_Godzinach”, Department of Acute Medicine, Faculty of Medical Science in Zabrze, Medical University of Silesia, 41-800 Zabrze, Poland; kzerdzinskik@gmail.com (K.Ż.); s86715@365.sum.edu.pl (M.G.); 2 Department of Biostatistics, Faculty of Public Health in Bytom, Medical University of Silesia in Katowice, 41-902 Bytom, Poland; mskrzypek@sum.edu.pl; 3 Department of Acute Medicine, Faculty of Medical Science in Zabrze, Medical University of Silesia, 41-800 Zabrze, Poland; lkrzych@sum.edu.pl; 4 Department of Anesthesiology and Intensive Care, Upper-Silesian Medical Center, 40-635 Katowice, Poland

**Keywords:** lactate, C-reactive protein, procalcitonin, albumin, intensive care unit, critical care, risk stratification, mortality, prognosis, biomarkers

## Abstract

**Background:** This study evaluates whether maximal and mean lactate-to-albumin (LAR), C-reactive protein-to-albumin (CAR), and procalcitonin-to-albumin (PAR) ratios during ICU stay improve mortality prediction beyond admission values in critically ill, cardiovascularly burdened patients, and compares their performance against individual biomarkers. **Methods:** We conducted a retrospective single-center cohort study of consecutive adult ICU admissions (2024) in a tertiary cardiac center, using temporally aligned (±12 h) albumin-based ratios derived from serial laboratory measurements. Maximal and mean in-ICU values were analysed per admission. The primary endpoint was ICU mortality. Associations and discriminative performance were evaluated using ROC analysis and multivariable logistic regression. **Results:** Of 212 screened ICU admissions, 137 patients were included. The ICU mortality was 48.9%. Both maximum and mean values of biomarkers and composite ratios were more abnormal in non-survivors, with the strongest independent associations observed for maximum LAR and CAR. In adjusted analyses, maximum LAR and CAR remained independently associated with ICU mortality and showed moderate discrimination (AUC 0.704 and 0.712). Mean CAR and LAR performed numerically slightly better in ROC analysis, whereas PAR showed weaker and less consistent prognostic utility. **Conclusions:** Maximal and mean LAR and CAR were independently associated with ICU mortality, whereas PAR showed weaker performance. These ratios may serve as simple tools for dynamic risk stratification but require prospective multicenter validation.

## 1. Introduction

Those patients who are in need of hospitalization in intensive care units (ICUs) remain at substantial risk of death despite advances in monitoring and organ support. This risk is further amplified in patients with significant cardiovascular comorbidity and multi-organ dysfunction, who constitute a growing proportion of contemporary ICU case-mix and often experience particularly poor short-term outcomes [1,2,3,4,5]. In such cardiovascularly burdened populations, early and pragmatic risk stratification is essential to support triage decisions, tailor treatment intensity, and inform prognostic discussions.

Although complex severity scores (e.g., APACHE, SOFA) are widely used upon ICU admission, they require multiple inputs and may be impractical, when ‘time-limited trial’ approach is applied [6,7]. Routine laboratory biomarkers offer a scalable alternative. Lactate (LAC) reflects impaired tissue perfusion and anaerobic metabolism, whereas C-reactive protein (CRP) and procalcitonin (PCT) capture systemic inflammation and infection-related responses; albumin (ALB), in turn, integrates chronic disease burden, nutritional status, and hepatic synthetic capacity [8,9,10,11,12]. However, each marker alone is susceptible to confounding and may incompletely reflect the combined acute-chronic vulnerability that characterizes critically ill, cardiovascularly burdened patients.

To address this limitation, composite ratios that normalize acute stress or inflammatory signals to ALB have gained attention. The lactate-to-albumin ratio (LAR), CRP-to-albumin ratio (CAR), and procalcitonin-to-albumin ratio (PAR) are designed to integrate an acute derangement (hypoperfusion, inflammation, or infection) with reduced physiological reserves, potentially strengthening the prognostic signal compared with their individual components [9,10,11,12,13,14,15]. Prior observational studies across heterogeneous ICU cohorts and selected high-risk groups suggest that these ratios are associated with mortality and may provide clinically useful discrimination.

Notably, the evidence base has focused predominantly on admission values. In a recent analysis from the same tertiary cardiac ICU cohort, we reported that early peri-admission LAR and CAR showed the most consistent association with ICU mortality, while PAR demonstrated weaker and less consistent performance [16]. However, peri-admission measurements primarily reflect initial severity and may not capture subsequent disease evolution and treatment response. In analogy to serial lactate assessment and lactate clearance, it is plausible that the intensity and persistence of derangements in LAR/CAR/PAR over the ICU course carry additional prognostic information. Maximal values may reflect the worst physiological disturbance and failure to reverse hypoperfusion or inflammatory burden, whereas mean values may approximate cumulative metabolic–inflammatory exposure. Despite this coherent rationale, the prognostic value of maximal and mean LAR, CAR, and PAR—particularly in critically ill adults with substantial cardiovascular comorbidity—remains poorly defined, and their comparative performance against the underlying single biomarkers is uncertain.

Therefore, in this complementary analysis, we evaluated the association of maximal LAR, CAR, and PAR values during ICU stay with ICU mortality in critically ill, cardiovascularly burdened adults. As secondary objectives, we compared the prognostic performance of maximal ratios with mean ratios and with their individual components (LAC, CRP, PCT, and ALB). We hypothesized that higher maximal LAR, CAR, and PAR would be independently associated with increased mortality and would demonstrate superior discrimination compared with mean ratios and single biomarkers in this high-risk population.

## 2. Methods

### 2.1. Study Design

A retrospective single-center observational cohort study including consecutive adult admissions to ICU between 1 January and 31 December 2024 was conducted. Clinical care during index ICU stays was routine and not influenced by the study procedures. Laboratory results (albumin, lactate, C-reactive protein, procalcitonin) and outcomes were retrospectively extracted from electronic records between 21 March and 28 July 2025.

### 2.2. Study Setting

The study was conducted in the ICU of the Silesian Center for Heart Diseases (Zabrze, Poland), a tertiary cardiac and cardiac-surgical referral center providing advanced cardiovascular critical care (including mechanical circulatory/respiratory support). The unit operates within the Medical University of Silesia structure.

### 2.3. Population, Inclusion and Exclusion Criteria

All adult patients (≥18 years) admitted between 1 January and 31 December 2024 were screened. Only the first eligible ICU admission per patient during the study period was analysed.

To enable derivation of composite indices, patients were included if at least one albumin (ALB) measurement during the ICU stay could serve as an anchoring time point for ratio calculation and if the corresponding biomarker (LAC for LAR, CRP for CAR, PCT for PAR) was measured within ±12 h of that ALB sampling. This approach ensured temporal proximity between numerator and denominator when calculating ratios and allowed computation of repeated ratio values across the ICU stay.

Exclusion criteria were: age < 18 years, absence of ALB measurement during ICU stay, lack of corresponding biomarker measurements within the prespecified ±12 h window, duplicate records, and ambiguous inter-ward transfers precluding reliable attribution of laboratory results to a given ICU stay.

Included ICU stays were categorized into six groups according to the leading diagnosis, as derived from individual discharge summaries (epicrises). Each case was independently classified by at least two investigators. In case of disagreement, a third reviewer adjudicated, and final allocation required concordance of at least two assessors. The diagnostic groups were defined as follows:

- CF (Cardiovascular Failure): Cardiogenic shock, ST-elevation myocardial infarction (STEMI), non-ST-elevation myocardial infarction (NSTEMI), post-cardiac surgery, and veno-arterial ECMO.
- CA (Post-Cardiac Arrest): Patients hospitalized due to multi-organ failure following cardiac arrest.
- RF (Respiratory Failure): Acute respiratory distress syndrome (ARDS), acute respiratory failure, pulmonary oedema, veno-venous ECMO, and severe pulmonary inflammation.
- VF (Vascular Failure): Patients hospitalized due to complications after vascular surgery.
- S (Sepsis): Sepsis of any origin.
- M (Mixed): Concomitant presence of more than one leading condition, not classified above.

### 2.4. Data Sources and Data Collection

Data were retrieved from the AMMS electronic medical record system integrated with the institutional laboratory information system. Extracted variables included age, sex, ICU admission/discharge timestamps, diagnostic category, ICU outcome (survival vs. death), ICU length of stay, and all available ALB, LAC, CRP and PCT measurements with sampling times between ICU admission and discharge. Records underwent deduplication and basic consistency checks (chronology and plausibility ranges). Flagged values were verified against source documentation. No formal statistical outlier exclusion algorithm was applied. Flagged values were retained if confirmed as genuine clinical measurements upon review of source documentation. The wide observed ranges for certain biomarkers—including maximum PCT of 311.44 μg/L and maximum LAC of 21.74 mmol/L—reflect genuine physiological extremes encountered in this high-acuity population and were not excluded from analyses. For quality assurance, 10% of randomly selected records were cross-checked against the original electronic documentation. Missing data were not imputed.

Of 212 screened admissions, 75 (35.4%) were excluded due to insufficient biomarker data: 63 (29.7%) lacked any albumin measurement during the ICU stay, 6 (2.8%) lacked a corresponding PCT measurement within the ±12 h window, 3 (1.4%) lacked CRP, and 1 (0.5%) lacked LAC. Two additional admissions were excluded due to incomplete discharge documentation. Among the 137 included patients, data for all four biomarkers were complete by design, as their availability constituted a formal inclusion criterion. Multiple imputation was therefore not applicable within the analysed cohort. The predominance of missing albumin measurements (29.7% of screened admissions) is unlikely to reflect random missingness, as discussed in the Section 4.

The working dataset was pseudonymized (coded IDs). The linkage key was stored separately in an encrypted offline file with restricted access.

### 2.5. Definitions and Units of the Indices

All laboratory parameters were expressed in the units listed in Table 1. On this basis, three albumin-based composite ratios were derived as follows:

$$LAR\;[\frac{mmol}{g}]=\frac{Lactate\;[mmol/L]}{Albumin\;[g/L]}$$

$$CAR\;[\frac{mg}{g}]=\frac{C-reactive\;protein\;[mg/L]}{Albumin\;[g/L]}$$

$$PAR\;[\frac{\mu g}{g}]=\frac{Procalcitonin\;[\mu g/L]}{Albumin\;[g/L]}$$

ALB measurements served as anchoring time points for ratio calculation. For each ALB sampling during the ICU stay, corresponding LAC, CRP and PCT values measured within a predefined ±12 h window relative to the ALB sampling time were identified. The highest available value was selected independently for each numerator biomarker (LAC, CRP, and PCT separately) within that window. Consequently, the LAC, CRP, and PCT values paired to the same ALB measurement may originate from different timestamps within the ±12 h interval, and a single ALB value may be paired with numerator values drawn from distinct time points. This approach was chosen to capture the peak acute physiological derangement available near each albumin anchor, consistent with the study’s focus on maximal stress exposure.

This procedure allowed multiple ratio values to be generated during a single ICU stay. For each admission, the maximum in-ICU value of each composite ratio (LAR, CAR, PAR) was used as the primary exposure variable. In secondary analyses, the mean in-ICU value of each ratio was calculated as the arithmetic mean of all ratio values derived during the ICU stay (i.e., the sum of all calculated ratio values divided by their number).

When analysed as standalone variables, single biomarkers (LAC, CRP, PCT, ALB) were evaluated using their maximum in-ICU values, whereas mean in-ICU values were calculated as the arithmetic mean of all available measurements for the respective biomarker during the ICU stay. All statistical analyses and ROC-derived cut-off values were performed using the units specified above.

### 2.6. Endpoints

The primary endpoint was ICU mortality, defined as death during the ICU stay. The primary analysis evaluated the association between ICU mortality and the maximum in-ICU values of the composite ratios (LAR, CAR, PAR). Secondary analyses included: assessment of ICU mortality in relation to mean in-ICU values of the composite ratios, evaluation of ICU mortality in relation to maximum and mean in-ICU values of the corresponding single biomarkers (LAC, CRP, PCT, ALB), and comparison of the discriminative performance (AUC) of composite ratios versus single biomarkers for ICU mortality.

### 2.7. Statistical Analysis

Statistical analysis was performed using R software, version 4.5.1 (R Foundation for Statistical Computing, Vienna, Austria). Quantitative variables with a normal distribution were presented as mean with standard deviation, whereas those deviating from normality were reported as median with lower and upper quartiles. Normality was assessed using the Shapiro–Wilk test. Categorical variables were described as absolute counts and percentages. Between-group comparisons for categorical variables were performed using the χ^2^ test. For quantitative variables, depending on distribution, Student’s *t*-test or Mann–Whitney U test was used for comparisons between two groups, and ANOVA or the Kruskal–Wallis test for comparisons across multiple groups. The predictive ability of the analysed variables for ICU mortality was assessed using the area under the receiver operating characteristic curve (AUC, Area Under the Curve). In addition, a multivariable logistic regression model was applied to estimate the risk of death, and the results were reported as odds ratios (OR) with 95% confidence intervals (CI). A *p*-value <0.05 was considered statistically significant.

The multivariable logistic regression models included up to ten parameters (age, sex, five admission category indicators, and up to four biomarker terms) against 67 outcome events, yielding an events-per-variable ratio of approximately 6–7, which falls below the commonly recommended threshold of 10. This reflects the inherent constraint of the available sample size. Adjustment was therefore intentionally parsimonious—limited to age, sex, and admission category—to reduce the risk of overfitting while retaining the clinically most relevant confounders. Residual confounding by unmeasured severity scores, comorbidities, and early ICU interventions cannot be excluded, as discussed in the Section 4.

As a pre-specified sensitivity analysis, the primary multivariable logistic regression models (Models 3 and 4) were re-run after excluding patients with extreme outlier values (defined as values exceeding Q3 + 3 × IQR) in at least one of the composite ratio numerators (LAC, CRP, or PCT) or in the composite ratios (LAR, CAR, or PAR), yielding sensitivity cohorts of *n* = 121 and *n* = 113, respectively. Full results are presented in Supplementary Table S1.

### 2.8. Use of Generative AI Tools

**Figure 1:**
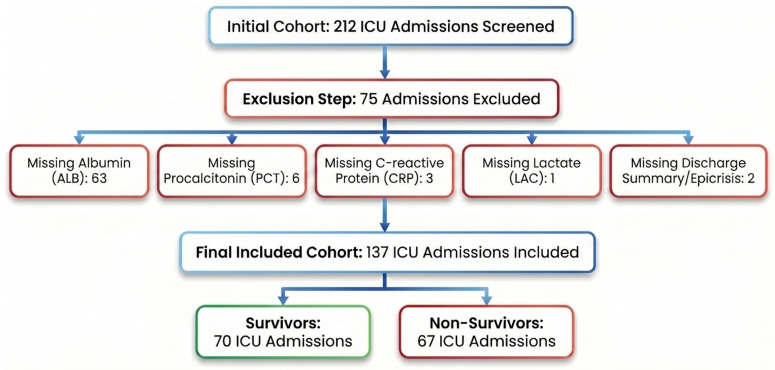
Flow of ICU admissions and availability of maximum LAR, CAR, and PAR values.

During the preparation of this manuscript, generative artificial intelligence tools were used in a strictly assistive capacity. ChatGPT (OpenAI, GPT-5 series) and Claude (Anthropic, Sonnet/Opus, v4.6/4.8) were employed during the study planning stage and for assistance in drafting section headings, figure and table captions, and language editing (spelling, grammar, and punctuation). Gemini (Google, v3) was used to generate the visual layout of Figure 1 under the supervision and full control of the authors. AI tools were not used for data collection, statistical analysis, interpretation of results, or scientific decision-making. All AI-assisted content was critically reviewed, edited, and validated for scientific accuracy by the authors prior to submission.

## 3. Results

Between 1 January and 31 December 2024, 212 ICU admissions were screened. After applying the predefined inclusion and exclusion criteria, 137 unique patients (one eligible ICU stay per patient) with at least one albumin-anchored time point enabling calculation of LAR, CAR, and PAR were included in the analysis (Figure 1). Because the primary analyses were based on the maximum in-ICU values of these indices, we summarized the number of available ratio calculation time points per patient (i.e., time points at which all three ratios could be computed). One complete LAR/CAR/PAR time point was available in 72 patients, while two and three time points were available in 21 and 22 patients, respectively. More than three time points were recorded in 22 patients.

### 3.1. Study Population and Baseline Characteristics

Among the 137 ICU stays included in the analysis, the median age was 69 years (IQR 55–75), 96 patients (70.1%) were male, and the median ICU LOS was 8 days (IQR 3–19). Cardiac failure and MOF post-cardiac arrest were the most frequent admission categories (36.5% and 23.4%, respectively), followed by respiratory (15.3%) and vascular (13.9%) pathologies, whereas sepsis and mixed indications were less common (5.8% and 5.1%). The ICU mortality was 48.9%, with 70 survivors and 67 non-survivors (Table 2).

Maximum and mean values of both simple and composite biomarkers showed wide ranges during the ICU stay (Table 3). Median maximum LAC, CRP, and PCT concentrations were 2.46, 150.31, and 2.27, respectively, while median minimum ALB was 32.0. The corresponding median maximum values of LAR, CAR, and PAR were 0.078, 5.292, and 0.073, with analogous distributions observed for mean values over the ICU course.

### 3.2. Clinical and Biomarker Profiles by ICU Survival Status

Among the 137 ICU stays, age, sex distribution, and admission categories were similar in ICU survivors and non-survivors, whereas survivors had a modestly longer ICU LOS (median 9.5 vs. 7 days; *p* = 0.048) (Table 4). This finding most plausibly reflects early death among non-survivors, who by definition had shorter stays, rather than a protective effect of prolonged ICU care. It is consistent with the survivorship structure inherent to this type of analysis and has been reported in comparable retrospective ICU cohorts.

Both maximum and mean values of simple biomarkers were consistently more abnormal in non-survivors, who had higher LAC, CRP, and PCT levels and lower ALB concentrations than survivors (all *p* ≤ 0.002). For example, median maximum LAC was 3.06 vs. 2.09 and median maximum CRP 190.63 vs. 108.59 in non-survivors versus survivors, while mean ALB was 28.69 vs. 32.25. Composite indices showed the same pattern: non-survivors had higher maximum and mean LAR, CAR, and PAR, with all between-group differences highly statistically significant (*p* < 0.001) (Table 5).

### 3.3. Associations Between Maximum Biomarker Values and ICU Mortality

In multivariable logistic regression models adjusted for age, sex and ICU admission category, selected maximum biomarker values were independently associated with ICU mortality, whereas neither sex nor admission category was significantly related to death in any model.

#### 3.3.1. Maximum Simple Biomarkers (LAC, CRP, PCT, ALB)

In the model incorporating age, sex, ICU admission category and maximum simple biomarkers, higher maximum LAC and CRP were independently associated with higher odds of ICU mortality (OR 1.157, 95% CI 1.043–1.304, *p* = 0.010, and OR 1.007, 95% CI 1.003–1.012, *p* = 0.002, respectively), whereas maximum PCT and minimum ALB were not significantly associated with outcome (Table 6). Increasing age was also associated with higher ICU mortality (OR 1.037, 95% CI 1.004–1.074, *p* = 0.034), while sex and ICU admission categories showed no statistically significant relationship with death.

#### 3.3.2. Maximum Composite Ratios (LAR, CAR, PAR)

In the corresponding model including age, sex, ICU admission category and maximum composite ratios, both maximum LAR and CAR remained independently associated with ICU mortality (OR 1.042, 95% CI 1.013–1.077, *p* = 0.007, and OR 1.252, 95% CI 1.114–1.425, *p* < 0.001, respectively), whereas maximum PAR was not significantly related to outcome (Table 7). Age again showed a modest but significant association with ICU mortality (OR 1.035, 95% CI 1.003–1.072, *p* = 0.038), while sex and ICU admission categories were not independent predictors of death in this model.

The primary associations were confirmed in pre-specified sensitivity analyses excluding extreme outlier values (Q3 + 3 × IQR applied independently to composite ratios): maximum LAR and CAR remained independently associated with ICU mortality (OR 1.095, 95% CI 1.032–1.178, *p* = 0.006 and OR 1.345, 95% CI 1.167–1.586, *p* < 0.001, respectively) in the sensitivity cohort of *n* = 113, with effect estimates consistent with or exceeding those observed in the primary analysis. Maximum PAR did not reach statistical significance in either cohort, consistent with the primary results. Full sensitivity analysis results are presented in Supplementary Table S1.

### 3.4. Discriminatory Performance of Maximum Ratios and Single Biomarkers: ROC Analyses and Cut-Off Values

#### 3.4.1. ROC Curves for Maximum Ratios and Single Biomarkers

**Figure 2:**
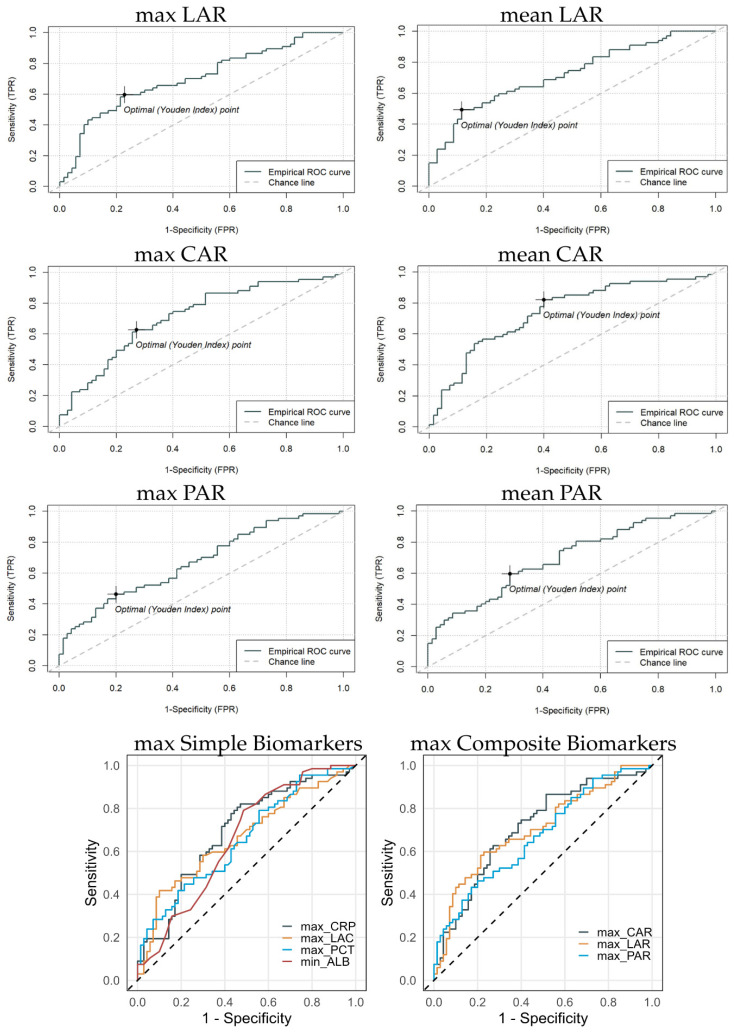
Receiver operating characteristic curve for maximum and mean LAR, CAR, PAR for ICU mortality.

In ROC analysis, maximum values of both single biomarkers and composite ratios demonstrated moderate discrimination between ICU survivors and non-survivors. Among composite indices, CAR and LAR yielded the highest AUC values (0.712 and 0.704, respectively), whereas PAR showed slightly lower discrimination (AUC 0.672) with reduced sensitivity at comparable specificity (Figure 2). CRP was the best-performing single biomarker, with an ROC curve similar to that of CAR and clearly superior to those of LAC, PCT, and ALB.

#### 3.4.2. Diagnostic Performance at ROC-Derived Cut-Offs

At cut-offs derived using the Youden index, maximum LAR and CAR achieved AUCs of 0.704 and 0.712, with accuracies of approximately 0.68 and sensitivities between 0.60 and 0.63. PAR showed a slightly lower AUC of 0.672, mainly due to reduced sensitivity (0.463) despite the highest specificity (0.80). Positive predictive values for all three ratios were around 0.69–0.71, and negative predictive values ranged from 0.61 to 0.67. Positive likelihood ratios for LAR, CAR, and PAR ranged from 2.31 to 2.61, whereas negative likelihood ratios were between 0.51 and 0.67 (Table 8).

At cut-offs derived using the Youden index, mean CAR and LAR achieved AUCs of 0.741 and 0.723, with accuracies of approximately 0.71 and 0.69, respectively. CAR showed the highest sensitivity (0.821) but lower specificity (0.60), whereas LAR demonstrated the opposite pattern, with high specificity (0.886) and reduced sensitivity (0.493). PAR yielded slightly lower discrimination (AUC 0.697) with balanced sensitivity and specificity (~0.60–0.71). Positive predictive values ranged from 0.66 to 0.81 and negative predictive values from 0.65 to 0.78. Positive likelihood ratios were highest for LAR (4.31), while for CAR and PAR they were around 2.05–2.09. Negative likelihood ratios ranged from 0.30 for CAR to approximately 0.56–0.57 for LAR and PAR (Table 9).

#### 3.4.3. The ICU Mortality Across Strata Defined by Maximum and Mean Ratio Cut-Offs

When patients were stratified according to these cut-offs, higher values of all three maximum ratios were associated with several-fold-higher odds of ICU mortality, with odds ratios of 5.00 for LAR, 4.51 for CAR, and 3.44 for PAR (all *p* ≤ 0.001). Mean ratio–based cut-offs showed an even stronger association, with odds ratios of 7.52 for LAR and 6.88 for CAR, while PAR again had a smaller but statistically significant effect (OR 3.70, *p* < 0.001). Overall, odds ratios were highest for LAR and CAR for both maximum and mean ratios (Table 10).

### 3.5. Subgroup Analyses

#### 3.5.1. The ICU Admission Category–Based Analyses of Maximum and Mean Biomarker Ratios

In analyses stratified by ICU admission category, ICU mortality did not differ significantly across groups (*p* = 0.10), although mortality proportions were numerically highest among patients admitted with sepsis (75.0%) and mixed indications (71.4%). Age and ICU LOS varied markedly between categories (both *p* < 0.001): patients with respiratory failure and sepsis were the youngest, whereas those with vascular pathology were oldest. The ICU stay was longest in respiratory failure (median 16 days) and shortest in vascular pathology (median 3 days).

Maximum and mean LAC values showed only a trend towards higher levels in selected categories (*p* = 0.068), whereas PCT and PAR differed significantly across admission groups (*p* = 0.013 and *p* = 0.019), with the highest median values observed in the sepsis category. Differences in LAR and CAR across admission categories were of smaller absolute magnitude compared with those observed in the survivor vs. non-survivor analysis (Table 5). Mean LAR reached statistical significance (*p* = 0.025), though this result should be interpreted cautiously in the context of multiple comparisons across subgroups (see Limitations). Mean CAR showed a non-significant trend (*p* = 0.065), with numerically higher values in sepsis and mixed admissions (Table 11).

#### 3.5.2. Sex-Specific Analyses of Maximum and Mean Biomarker Values

The ICU mortality, ICU LOS, and age did not differ significantly between women and men (all *p* ≥ 0.3). Maximum and mean values of simple biomarkers and composite ratios were also broadly similar across sexes. Men had numerically higher CRP and CAR values, particularly mean CAR (median 4.61 vs. 2.81; *p* = 0.081), but none of these differences reached statistical significance (Table 12).

### 3.6. Spearman Correlations Between Maximum Composite Ratios and Clinical Variables

In Spearman correlation analysis, maximum LAR, CAR, and PAR showed only weak associations with basic demographics. The strongest demographic relationship was a modest inverse correlation between age and maximum PAR (rho = −0.24, *p* = 0.004), whereas correlations with sex were close to zero (|rho| ≤ 0.12). By contrast, composite ratios were positively correlated with one another, with rho = 0.22 for LAR–CAR, 0.38 for LAR–PAR, and 0.56 for CAR–PAR (all *p* < 0.01) (Table 13).

## 4. Discussion

This study assessed the prognostic value of maximum and mean in-ICU LAR, CAR, and PAR in critically ill, cardiovascularly burdened adults, extending our prior analysis of peri-admission measurements from the same cohort [16]. The central finding is that dynamic in-ICU assessment of LAR and CAR—capturing both peak severity and cumulative exposure—provided stronger and more consistent prognostic associations with ICU mortality than static admission-time values, while PAR showed weaker and less reliable performance throughout. Most prior evidence has relied on single-timepoint biomarker assessments. The present analysis contributes evidence that the intensity and persistence of composite ratio derangements across the ICU course carry additional prognostic information beyond the early admission snapshot.

The diagnostic utility of albumin-based composite indices in this population stems from their ability to simultaneously reflect acute physiological stress and baseline metabolic reserve [8,16,17,18,19,20,21,22]. In patients with significant cardiovascular burden, individual biomarkers may be insufficient in isolation: lactate alone does not capture protein reserve, and albumin alone does not quantify the acute perfusion signal. By normalising lactate, CRP, or procalcitonin to albumin, a parameter is obtained that integrates acute derangement with chronic vulnerability—potentially identifying patients in whom even moderate elevations in inflammatory or metabolic markers, against a background of severely depleted protein reserves, herald critical homeostatic failure. The consistently more abnormal serial biomarker profiles observed in non-survivors across all three composite indices are consistent with this rationale and align with the growing evidence base for albumin-based ratios in heterogeneous and cardiac ICU populations [17,18,19,20,21,22].

LAR captures the combined signal of tissue hypoperfusion and nutritional-metabolic reserve. Lactate reflects the depth of oxygen debt. In a cardiovascularly burdened population, hyperlactataemia is a direct indicator of circulatory failure, and failure of lactate clearance in response to resuscitation is a well-established marker of treatment failure [18]. Capillary leak syndrome further exacerbates this signal by driving albumin into the extravascular space, accentuating LAR elevation in the most critically ill patients [22]. In the present study, maximum LAR (AUC 0.704) and mean LAR (AUC 0.723) showed numerically higher discrimination than peri-admission LAR from our prior analysis of the same cohort (AUC 0.692 [16]), suggesting that capturing the peak or cumulative derangement adds prognostic information beyond the early measurement alone.

CAR integrates CRP—which rises rapidly in response to ischaemia, infection, and tissue damage and correlates with organ failure burden [21]—with albumin, simultaneously quantifying inflammatory aggression and physiological reserve. In the present study, maximum CAR (AUC 0.712) and mean CAR (AUC 0.741) were the strongest discriminators among the three composite indices, and maximum CAR showed the strongest and most consistent association with ICU mortality in adjusted models. These findings extend our prior observation that peri-admission CAR (AUC 0.677 [16]) was independently associated with ICU death, and are consistent with reports demonstrating superiority of peak CAR values over admission-time indices in cardiac patients [23]. The numerically higher AUC for mean CAR over maximum CAR is mechanistically plausible: mean CAR captures cumulative inflammatory exposure across the ICU stay, whereas maximum CAR identifies the single moment of greatest imbalance.

PAR combines PCT—a marker with recognized specificity for bacterial infection and sepsis—with albumin. In this cohort, which was overwhelmingly cardiovascular in aetiology with sepsis representing only 5.8% of admissions, PAR showed the weakest and least consistent performance: maximum PAR yielded AUC 0.672 and was not an independent predictor in the adjusted model, while mean PAR (AUC 0.697) showed only modest improvement. This is consistent with our prior admission-time analysis (PAR AUC 0.625 [16]) and may reflect the context-dependence of PCT in cardiac ICU populations, where non-infectious PCT elevations—such as those arising from bacterial translocation during cardiogenic shock—may attenuate prognostic specificity [20].

Placing the present findings alongside our prior publication [16] allows a structured inspection of the AUC trajectory across measurement strategies in the same cohort: for LAR, AUCs were 0.692 (peri-admission), 0.704 (maximum in-ICU), and 0.723 (mean in-ICU); for CAR, 0.677, 0.712, and 0.741; and for PAR, 0.625, 0.672, and 0.697. In each case, mean in-ICU values yielded numerically higher AUCs than either maximum values or peri-admission values, suggesting that integrating the full in-ICU biomarker trajectory may provide a stronger prognostic signal than a single early or extreme measurement. However, formal pairwise AUC comparisons using DeLong’s method were not performed, and the observed differences cannot be confirmed as statistically significant. They should therefore be treated as descriptive and hypothesis-generating. Future prospective studies should pre-specify formal AUC comparison methodology and include head-to-head comparisons against admission-only models and established severity scores.

The practical implication is that serial calculation of LAR and CAR from routine laboratory measurements—already obtained as standard ICU care—may support dynamic risk stratification during hospitalization. A rise in these indices toward peak values signals potential treatment failure, persistent hypoperfusion, or insufficient inflammatory control. A declining trajectory may support progressive titration of treatment intensity. This approach conceptually aligns with established serial lactate monitoring [18] and extends it to albumin-normalized composite parameters. Whether integration of dynamic LAR and CAR into clinical practice or electronic decision-support tools improves outcomes beyond conventional assessment requires prospective, multicenter evaluation with formal clinical utility analysis.

The main limitation of this study is its retrospective, single-center design and the specific cohort of cardiovascularly burdened patients admitted to a tertiary cardiac ICU, which constrains the generalizability of the derived cut-off values to the broader ICU population. The strict ±12 h temporal alignment window for albumin anchoring limited the analyzable sample to 137 patients. Notably, 63 of 212 screened admissions were excluded due to missing albumin measurements, representing 29.7% of the screened cohort. Missingness of albumin is unlikely to be random in an ICU context, as ordering patterns may vary with presentation acuity, pre-ICU workup location, early death, or clinical instability. Accordingly, included patients may over-represent those with more complete early laboratory monitoring, and the transportability of derived cut-offs to settings with lower albumin measurement rates cannot be assumed.

No formal statistical outlier exclusion algorithm was applied. Extreme values were individually verified against source documentation and retained if confirmed as genuine clinical measurements. A pre-specified sensitivity analysis excluding patients with extreme outlier values (Q3 + 3 × IQR, applied independently to composite ratios LAR, CAR, and PAR; *n* = 113) confirmed the robustness of the primary findings, with maximum LAR and CAR remaining independently associated with ICU mortality (Supplementary Table S1). The Q3 + 3 × IQR threshold represents one of several defensible criteria for extreme outlier identification; alternative thresholds could yield marginally different sensitivity cohorts, and the applied criterion should not be interpreted as the uniquely valid approach.

Validated ICU severity scores (SOFA, APACHE II, SAPS II), granular comorbidity histories (chronic kidney disease, liver disease, malignancy), and specific organ support parameters (mechanical ventilation, vasopressor exposure, renal replacement therapy) were not systematically available in a uniform, extractable format across the full cohort. Reliable retrospective reconstruction was not feasible without introducing additional missingness and misclassification, as previously noted in our analysis of the same cohort [16]. To avoid collinearity, composite ratios and their constituent biomarkers were not simultaneously included in a single multivariable model, which precludes direct incremental value testing of ratios over components in a unified specification. Residual confounding by unmeasured severity, comorbidity burden, and early ICU interventions cannot be excluded.

The timing and appropriateness of antimicrobial therapy and source control were not systematically tracked as discrete variables. Variations in these interventions may have independently influenced the peak and mean values of CRP and PCT, and consequently of CAR and PAR, introducing unquantifiable confounding in the inflammation-based ratios—particularly relevant for the sepsis and mixed subgroups, despite their small representation in this cohort. Additionally, the analysis does not account for the dilutional effect of aggressive fluid resuscitation on albumin concentration, which may have influenced the denominator of all three indices. Finally, the varying frequency of serial laboratory sampling—driven by clinical need and length of stay rather than a standardized protocol—may have affected the precision of the estimated maximum and mean in-ICU values, particularly in patients with short ICU stays or early discharge.

Several additional analytical limitations should be acknowledged. First, pairwise AUC comparisons between maximum and mean ratio values, and between composite ratios and their individual biomarker components, were not performed using formal statistical methods (e.g., DeLong’s test). Accordingly, observed AUC differences between strategies—such as 0.704 versus 0.723 for LAR—should be treated as descriptive and may reflect chance rather than true discrimination differences. Second, a sensitivity analysis substituting the median (rather than the maximum) numerator biomarker value within the ±12 h alignment window was not conducted. It is therefore uncertain whether the use of maximum values systematically inflated ratio estimates or overstated prognostic associations compared with a more conservative central-tendency selection rule. Third, no correction for multiple comparisons (e.g., Benjamini–Hochberg or Bonferroni) was applied to subgroup analyses in Table 11; with eleven simultaneous comparisons, the risk of spurious findings is non-trivial, and associations with *p*-values between 0.01 and 0.05 in this table should be interpreted as exploratory and hypothesis-generating rather than confirmatory. Future prospective studies should pre-specify AUC comparison methodology, outlier and window-selection sensitivity analyses, and multiple comparison correction strategies prior to data collection.

## 5. Conclusions

In cardiovascularly burdened adults, both maximal and mean LAR and CAR ratios were independently associated with ICU mortality after adjustment for age, sex, and admission category, with findings confirmed in a pre-specified sensitivity analysis. By contrast, the PAR showed only modest discrimination. Overall, albumin-based ratios emerge as simple, no-additional-cost adjuncts for dynamic risk stratification that can be repeatedly calculated from laboratory measurements already obtained as part of routine ICU care, without requiring additional sampling or dedicated assays. Prospective multicenter studies are needed to validate optimal cut-offs, clarify added value over conventional severity scores, and test integration of dynamic LAR and CAR into multimarker prediction models and electronic decision-support tools.

## Figures and Tables

**Table 1:** Reference laboratory ranges and units of collected data.

Reference Range and Units	
**LAC**	0.5–2.0 mmol/L
**CRP**	<5 mg/L
**PCT**	<0.5 μg/L
**ALB**	32–48 g/L

Abbreviations: LAC—Lactate; CRP—C-reactive protein; PCT—Procalcitonin; ALB—Albumin.

**Table 2:** Baseline demographic and clinical characteristics of the ICU cohort.

Characteristic		*n* = 137 ^1^
Age		69 (55, 75)
ICU LOS		8 (3, 19)
Sex	Female	41 (29.9%)
	Male	96 (70.1%)
Groups	Cardiovascular Failure	50 (36.5%)
	Post-Cardiac Arrest	32 (23.4%)
	Respiratory Failure	21 (15.3%)
	Vascular Failure	19 (13.9%)
	Sepsis	8 (5.8%)
	Mixed	7 (5.1%)
ICU mortality	Survivor	70 (51.1%)
	Non-survivor	67 (48.9%)

^1^ *n* (%); Median (Q1, Q3); Mean (SD); ICU—Intensive Care Unit; LOS—Length of stay.

**Table 3:** Maximum and mean values of simple and composite biomarkers during ICU stay.

Characteristic		Min	Q1	Median	Q3	Max
Maximum Values	LAC	0.700	1.720	2.460	5.030	21.740
	CRP	0.500	72.620	150.310	225.450	512.480
	PCT	0.030	0.600	2.270	8.140	311.440
	ALB ^1^	19.00	28.00	32.00	36.00	45.00
	LAR	0.0242	0.0541	0.078	0.1610	0.7500
	CAR	0.0139	2.5262	5.2920	8.5480	19.3900
	PAR	0.0007	0.0216	0.073	0.316	11.530
Mean Values	LAC	0.7000	1.5700	2.090	3.310	21.740
	CRP	0.5000	55.1100	115.377	178.012	377.470
	PCT	0.0300	0.5300	1.583	5.340	311.440
	ALB	19.00	26.60	30.00	34.00	45.00
	LAR	0.0242	0.0471	0.0710	0.1210	0.7500
	CAR	0.0139	2.0900	4.1620	6.3970	16.6500
	PAR	0.0007	0.0167	0.0570	0.1620	11.5300

^1^ Minimum value; LAR—Lactate-to-Albumin Ratio; CAR—C-Reactive Protein-to-Albumin Ratio; PAR—Procalcitonin-to-Albumin Ratio.

**Table 4:** Baseline demographic and clinical characteristics by ICU survival status.

Characteristic		Overall ^1^	Survivor ^1^	Non-Survivor ^1^	*p*-Value ^2^
Age		69 (55, 75)	69 (51, 76)	68 (58, 74)	0.8
ICU LOS		8 (3, 19)	9.5 (5, 22)	7 (3, 16)	0.048
Sex	Female	41 (29.9%)	21 (30.0%)	20 (29.9%)	>0.9
	Male	96 (70.1%)	49 (70.0%)	47 (70.1%)	
Groups	Cardiovascular Failure	50 (36.5%)	25 (35.7%)	25 (37.3%)	0.1
	Post-Cardiac Arrest	32 (23.4%)	15 (21.4%)	17 (25.4%)	
	Respiratory Failure	21 (15.3%)	16 (22.9%)	5 (7.5%)	
	Vascular Failure	19 (13.9%)	10 (14.3%)	9 (13.4%)	
	Sepsis	8 (5.8%)	2 (2.9%)	6 (9.0%)	
	Mixed	7 (5.1%)	2 (2.9%)	5 (7.5%)	

^1^ *n* (%); Median (Q1, Q3); Mean (SD); ^2^ Pearson’s Chi-squared test; Fisher’s exact test; Wilcoxon rank sum test; Welch Two Sample *t*-test.

**Table 5:** Maximum and mean simple and composite biomarker values by ICU survival status.

Characteristic		Overall ^1^	Survivor ^1^	Non-Survivor ^1^	*p*-Value ^2^
Maximum Values	LAC	2.46 (1.72, 5.03)	2.09 (1.56, 3.17)	3.06 (1.89, 7.30)	<0.001
	CRP	150.31 (72.62, 225.45)	108.59 (50.24, 195.77)	190.63 (126.81, 248.48)	<0.001
	PCT	2.27 (0.60, 8.14)	1.56 (0.28, 5.27)	3.27 (1.10, 16.27)	0.002
	ALB ^3^	32.09 (5.72)	33.84 (5.55)	30.27 (5.34)	<0.001
	LAR	0.08 (0.05, 0.16)	0.07 (0.05, 0.10)	0.12 (0.06, 0.26)	<0.001
	CAR	5.29 (2.53, 8.55)	3.74 (1.23, 6.41)	6.66 (4.22, 9.91)	<0.001
	PAR	0.07 (0.02, 0.32)	0.05 (0.01, 0.14)	0.11 (0.04, 0.67)	<0.001
Mean Values	LAC	2.09 (1.57, 3.31)	1.78 (1.41, 2.49)	2.68 (1.75, 5.43)	<0.001
	CRP	115.38 (55.11, 178.01)	80.44 (39.32, 150.58)	153.46 (103.85, 215.65)	<0.001
	PCT	1.58 (0.53, 5.34)	0.99 (0.27, 2.98)	2.30 (1.00, 9.13)	<0.001
	ALB	30.51 (5.22)	32.25 (5.47)	28.69 (4.28)	<0.001
	LAR	0.07 (0.05, 0.12)	0.06 (0.04, 0.08)	0.10 (0.06, 0.20)	<0.001
	CAR	4.16 (2.09, 6.40)	2.50 (1.14, 4.86)	5.72 (3.55, 7.06)	<0.001
	PAR	0.06 (0.02, 0.16)	0.03 (0.01, 0.09)	0.08 (0.03, 0.42)	<0.001

^1^ *n* (%); Median (Q1, Q3); Mean (SD); ^2^ Pearson’s Chi-squared test; Fisher’s exact test; Wilcoxon rank sum test; Welch Two Sample *t*-test; ^3^ Minimum value.

**Table 6:** Multivariable logistic regression of maximum simple biomarker values for ICU mortality.

Characteristic		OR	95% CI	*p*-Value
Age		1.037	1.004, 1.074	0.034
Sex	Female	—	—	
	Male	0.801	0.326, 1.944	0.625
Simple biomarkers	LAC	1.157	1.043, 1.304	0.010
	CRP	1.007	1.003, 1.012	0.002
	PCT	1.015	0.992, 1.052	0.295
	ALB ^1^	0.935	0.859, 1.014	0.112
Groups	Cardiovascular Failure	0.697	0.087, 4.016	0.700
	Post-Cardiac Arrest	1.215	0.137, 8.204	0.847
	Respiratory Failure	0.261	0.025, 1.964	0.214
	Vascular Failure	0.643	0.070, 4.469	0.666
	Sepsis	3.653	0.247, 58.712	0.339
	Mixed	—	—	

Abbreviations: CI—Confidence Interval, OR—Odds Ratio; ^1^ Minimum value.

**Table 7:** Multivariable logistic regression of maximum composite biomarker ratios for ICU mortality.

Characteristic		OR	95% CI	*p*-Value
Age		1.035	1.003, 1.072	0.038
Sex	Female	—	—	
	Male	0.765	0.315, 1.832	0.548
Composite biomarkers	LAR	1.042	1.013, 1.077	0.007
	CAR	1.252	1.114, 1.425	<0.001
	PAR	1.402	0.758, 3.453	0.368
Groups	Cardiovascular Failure	0.872	0.107, 5.218	0.886
	Post-Cardiac Arrest	1.232	0.140, 8.273	0.835
	Respiratory Failure	0.282	0.027, 2.126	0.240
	Vascular Failure	0.726	0.077, 5.232	0.758
	Sepsis	4.032	0.259, 69.080	0.314
	Mixed	—	—	

**Table 8:** Discriminatory performance of maximum LAR, CAR, and PAR for ICU mortality at Youden-index-derived cut-offs.

Marker	Cut-Off	AUC	Accuracy	Sensitivity	Specificity
LAR	0.1031	0.704 (0.616–0.791)	0.686 (0.628–0.766)	0.597 (0.47–0.715)	0.771 (0.656–0.863)
CAR	5.8363	0.712 (0.625–0.799)	0.679 (0.628–0.766)	0.627 (0.5–0.742)	0.729 (0.609–0.828)
PAR	0.1773	0.672 (0.583–0.762)	0.635 (0.599–0.715)	0.463 (0.34–0.589)	0.8 (0.687–0.886)
**Marker**	**Cut-Off**	**PPV**	**NPV**	**PDLR**	**NDLR**
LAR	0.1031	0.714 (0.585–0.809)	0.667 (0.545–0.789)	2.612 (1.627–4.192)	0.522 (0.38–0.718)
CAR	5.8363	0.689 (0.562–0.791)	0.671 (0.548–0.785)	2.31 (1.508–3.536)	0.512 (0.364–0.721)
PAR	0.1773	0.689 (0.549–0.786)	0.609 (0.482–0.752)	2.313 (1.355–3.95)	0.672 (0.522–0.863)

Abbreviations: AUC—Area Under the Curve; PPV Positive Predictive Value; NPV—Negative Predictive Value; PDLR—Positive Diagnostic Likelihood Ratio; NDLR—Negative Diagnostic Likelihood Ratio.

**Table 9:** Discriminatory performance of mean LAR, CAR, and PAR for ICU mortality at Youden-index-derived cut-offs.

Marker	Cut-Off	AUC	Accuracy	Sensitivity	Specificity
LAR	0.1064	0.723 (0.638–0.807)	0.693 (0.635–0.766)	0.493 (0.368–0.618)	0.886 (0.787–0.949)
CAR	3.3581	0.741 (0.657–0.825)	0.708 (0.657–0.788)	0.821 (0.708–0.904)	0.6 (0.476–0.715)
PAR	0.0652	0.697 (0.609–0.784)	0.657 (0.613–0.737)	0.597 (0.47–0.715)	0.714 (0.594–0.816)
**Marker**	**Cut-Off**	**PPV**	**NPV**	**PDLR**	**NDLR**
LAR	0.1064	0.805 (0.663–0.873)	0.646 (0.523–0.815)	4.31 (2.149–8.644)	0.573 (0.446–0.736)
CAR	3.3581	0.663 (0.543–0.801)	0.778 (0.649–0.854)	2.052 (1.508–2.792)	0.299 (0.173–0.516)
PAR	0.0652	0.667 (0.539–0.772)	0.649 (0.526–0.767)	2.09 (1.374–3.178)	0.564 (0.407–0.782)

Abbreviations: AUC—Area Under the Curve; PPV Positive Predictive Value; NPV—Negative Predictive Value; PDLR—Positive Diagnostic Likelihood Ratio; NDLR—Negative Diagnostic Likelihood Ratio.

**Table 10:** ICU mortality odds across strata defined by Youden-index-based cut-offs of maximum and mean LAR, CAR, and PAR.

Characteristic	Marker	Cut-Off	OR	95% CI	*p*-Value
Maximum values	LAR	0.1031	5.000	2.423, 10.73	<0.001
	CAR	5.8363	4.509	2.219, 9.464	<0.001
	PAR	0.1773	3.444	1.641, 7.519	0.001
Mean values	LAR	0.1064	7.522	3.256, 19.20	<0.001
	CAR	3.3581	6.875	3.211, 15.61	<0.001
	PAR	0.0652	3.704	1.838, 7.671	<0.001

All odds ratios are derived from univariable analyses stratified by the Youden-index-derived cut-off and are not adjusted for age, admission category, or other potential confounders. Abbreviations: CI—Confidence Interval; OR—Odds Ratio.

**Table 11:** Maximum and mean biomarker ratios, age, sex distribution, ICU LOS, and mortality across ICU admission categories.

Characteristic		Overall (*n* = 137) ^1^	CF (*n* = 50) ^1^	CA (*n* = 32) ^1^	RF (*n* = 21) ^1^	VF (*n* = 19) ^1^	S (*n* = 8) ^1^	M (*n* = 7) ^1^	*p*-Value ^2^
ICU mortality		67 (48.9%)	25 (50.0%)	17 (53.1%)	5 (23.8%)	9 (47.4%)	6 (75.0%)	5 (71.4%)	0.10
ICU LOS		8 (3, 19)	12 (4, 20)	8 (3, 11)	16 (7, 28)	3 (1, 6)	6.5 (3.5, 47)	9 (3, 21)	<0.001
Age		69 (55, 75)	69.50 (58, 76)	68 (60, 76)	52 (41, 64)	73 (68, 76)	54.5 (41, 63)	72 (62, 76)	<0.001
Sex	Female	41 (29.9%)	15 (30.0%)	8 (25.0%)	8 (38.1%)	6 (31.6%)	2 (25.0%)	2 (28.6%)	>0.9
	Male	96 (70.1%)	35 (70.0%)	24 (75.0%)	13 (61.9%)	13 (68.4%)	6 (75.0%)	5 (71.4%)	
Maximum values	LAC	2.46 (1.72, 5.03)	2.72 (1.78, 5.38)	2.96 (1.90, 7.32)	1.75 (1.57, 2.42)	2.07 (1.58, 7.34)	3.42 (1.96, 6.16)	2.99 (1.45, 7.30)	0.068
	CRP	150.31 (72.62, 225.45)	147.53 (77.67, 237.54)	134.27 (41.55, 209.22)	160.34 (69.26, 275.39)	147.57 (43.71, 171.17)	196.61 (62.70, 290.16)	210.26 (159.17, 227.64)	0.2
	PCT	2.27 (0.60, 8.14)	2.68 (0.73, 7.65)	2.36 (0.84, 8.29)	2.62 (0.99, 7.83)	0.45 (0.07, 4.11)	12.45 (5.55, 35.40)	2.09 (0.41, 9.70)	0.013
	ALB ^3^	28.96 (5.88)	28.18 (4.75)	30.97 (7.17)	30.24 (5.96)	28.53 (6.06)	26.75 (5.04)	25.14 (4.45)	0.11
	LAR	0.08 (0.05, 0.16)	0.10 (0.06, 0.16)	0.08 (0.06, 0.24)	0.06 (0.04, 0.08)	0.07 (0.05, 0.33)	0.12 (0.06, 0.29)	0.11 (0.06, 0.24)	0.12
	CAR	5.29 (2.53, 8.55)	5.25 (2.61, 8.34)	4.40 (1.01, 7.23)	6.01 (2.66, 9.47)	4.22 (2.30, 6.54)	7.34 (2.47, 12.31)	7.59 (7.24, 10.34)	0.14
	PAR	0.07 (0.02, 0.32)	0.08 (0.02, 0.33)	0.08 (0.03, 0.22)	0.06 (0.04, 0.25)	0.01 (0.00, 0.11)	0.51 (0.18, 1.59)	0.07 (0.02, 0.33)	0.019
Mean values	LAR	0.07 (0.05, 0.12)	0.08 (0.05, 0.11)	0.07 (0.06, 0.19)	0.05 (0.04, 0.06)	0.07 (0.05, 0.18)	0.12 (0.05, 0.19)	0.11 (0.04, 0.24)	0.025
	CAR	4.16 (2.09, 6.40)	3.95 (2.19, 5.81)	3.15 (1.01, 4.94)	4.45 (2.24, 6.80)	4.16 (2.30, 6.34)	6.34 (2.47, 10.52)	6.40 (5.51, 6.78)	0.065
	PAR	0.06 (0.02, 0.16)	0.05 (0.02, 0.11)	0.06 (0.02, 0.20)	0.06 (0.03, 0.13)	0.01 (0.00, 0.11)	0.40 (0.18, 1.17)	0.07 (0.02, 0.21)	0.026

^1^ *n* (%); Median (Q1, Q3); Mean (SD); ^2^ Fisher’s exact test; Kruskal–Wallis rank sum test; One-way analysis of means (not assuming equal variances); ^3^ Minimum value; Groups: CF—Cardiovascular Failure; CA—Post-Cardiac Arrest; RF—Respiratory Failure; VF—Vascular Failure; S—Sepsis; M—Mixed.

**Table 12:** Sex-specific comparison of maximum and mean simple biomarkers and composite ratios, age, ICU LOS, and mortality.

Characteristic		Overall (*n* = 137) ^1^	Female (*n* = 41) ^1^	Male (*n* = 96) ^1^	*p*-Value ^2^
ICU mortality		67 (48.9%)	20 (48.8%)	47 (49.0%)	>0.9
ICU LOS		8 (3, 19)	8 (4, 15)	8.5 (3, 20)	0.8
Age		69 (55, 75)	72 (47, 77)	67.5 (57, 73.5)	0.3
Maximum values	LAC	2.46 (1.72, 5.03)	2.84 (2.03, 3.67)	2.37 (1.67, 5.82)	0.4
	CRP	150.31 (72.62, 225.45)	118.33 (60.20, 210.36)	158.57 (76.63, 230.00)	0.2
	PCT	2.27 (0.60, 8.14)	2.27 (0.54, 8.14)	2.31 (0.60, 8.25)	0.7
	ALB ^3^	28.96 (5.88)	28.66 (4.69)	29.08 (6.34)	0.7
	LAR	0.08 (0.05, 0.16)	0.10 (0.06, 0.14)	0.08 (0.05, 0.18)	0.4
	CAR	5.29 (2.53, 8.55)	4.03 (2.30, 7.70)	5.69 (2.72, 9.14)	0.2
	PAR	0.07 (0.02, 0.32)	0.07 (0.02, 0.20)	0.08 (0.02, 0.35)	0.7
Mean values	LAR	0.07 (0.05, 0.12)	0.08 (0.06, 0.11)	0.07 (0.05, 0.12)	0.3
	CAR	4.16 (2.09, 6.40)	2.81 (1.73, 5.10)	4.61 (2.29, 6.58)	0.081
	PAR	0.06 (0.02, 0.16)	0.04 (0.02, 0.16)	0.06 (0.02, 0.17)	0.7

^1^ *n* (%); Median (Q1, Q3); Mean (SD); ^2^ Fisher’s exact test; Wilcoxon rank sum test; Pearson’s Chi-squared test; Welch Two Sample *t*-test; ^3^ Minimum value.

**Table 13:** Spearman correlation matrix for maximum LAR, CAR, and PAR with age, sex, and between-ratio correlations.

	Age	Sex	LAR	CAR	PAR
Age	1.000	−0.089	−0.062	−0.170	−0.240
Sex	−0.089	1.000	−0.079	0.120	0.035
LAR	−0.062	−0.079	1.000	0.220	0.380
CAR	−0.170	0.120	0.220	1.000	0.560
PAR	−0.240	0.035	0.380	0.560	1.000

Background color intensity reflects the strength and direction of the correlation coefficient (red = positive, blue = negative), consistent with the default heatmap output of R (v4.5.1).

## Data Availability

The data presented in this study are available from the scientific supervisor (Ł.J.K.) of the project upon reasonable request.

## Supplementary Materials

The following supporting information can be downloaded at https://www.mdpi.com/article/10.3390/jcm15124698/s1, Supplementary Material Table S1: Sensitivity analyses of primary multivariable logistic regression models for ICU mortality after exclusion of patients with extreme outlier values.

## Abbreviations

LAR	Lactate-to-albumin ratio
CAR	C-Reactive protein-to-albumin ratio
PAR	Procalcitonin-to-albumin ratio
ICU	Intensive care unit
LAC	Lactate
CRP	C-Reactive protein
PCT	Procalcitonin
ALB	Albumin
CF	Cardiovascular failure (group)
CA	Post-cardiac arrest and hospitalized due to its complications (group)
RF	Respiratory failure (group)
VF	Vascular failure (group)
S	Sepsis (group)
M	Mixed (group)
LOS	Length of stay
AUC	Area under the curve
OR	Odds ratios
CI	Confidence intervals
