# Supplementary material for: Prognostic Value of Maximal and Mean Lactate-to-Albumin (LAR), C-Reactive Protein-to-Albumin (CAR), and Procalcitonin-to-Albumin (PAR) Ratios Beyond ICU Admission in Critically Ill Patients
Source: J Clin Med. 2026 Jun 17;15(12):4698. doi: 10.3390/jcm15124698 (PMC13301477; doi:10.3390/jcm15124698)
Supplement: Supplementary file 1 [file jcm-15-04698-s001.zip › jcm-4299078-supplementary.pdf]

Supplementary Materials

Prognostic Value of Maximal and Mean Lactate-to-Albumin (LAR), C-Reactive Protein-to-Albumin (CAR), and Procalcitonin-to-Albumin (PAR) Ratios Beyond ICU Admission in Critically Ill Patients

Krzysztof Żerdziński [1,†], Michał Gałuszewski [1,†], Julita Janiec [1,*], Michał Skrzypek [2] and Łukasz J. Krzych [3,4]

Supplementary Table S1. Sensitivity analyses of primary multivariable logistic regression models for ICU mortality after exclusion of patients with extreme outlier values.

Panel A. Model including maximum simple biomarkers (Extreme outliers excluded: n = 121)

Characteristic	OR	95% CI	p-value
Age	1.036	1.001 – 1.077	0.055
**Sex**			
Female	—	—	—
Male	0.610	0.228 – 1.592	0.316
**Simple biomarkers**			
Maximum LAC	1.246	1.079 – 1.482	0.006
Maximum CRP	1.008	1.003 – 1.013	0.001
Maximum PCT	0.977	0.905 – 1.053	0.540
Minimum ALB	0.912	0.829 – 0.996	0.047
**Admission Category**			
Mixed	—	—	—
Cardiovascular Failure	0.728	0.087 – 4.489	0.742
Post-Cardiac Arrest	1.794	0.186 – 13.881	0.583
Respiratory Failure	0.284	0.026 – 2.267	0.254

Vascular Failure	0.850	0.087 – 6.411	0.878
Sepsis	4.340	0.258 – 83.489	0.306

Panel B. Model including maximum composite ratios (Extreme outliers excluded: n = 113)

**Characteristic**	**OR**	**95% CI**	**p-value**
Age	1.039	1.002 – 1.083	0.051
**Sex**			
Female	—	—	—
Male	0.587	0.213 – 1.572	0.292
**Composite biomarkers**			
Maximum LAR (per 0.01 units)	1.095	1.032 – 1.178	0.006
Maximum CAR	1.345	1.167 – 1.586	<0.001
Maximum PAR	0.577	0.053 – 6.631	0.648
**Admission Category**			
Mixed	—	—	—
Cardiovascular Failure	1.133	0.124 – 8.085	0.903
Post-Cardiac Arrest	3.034	0.292 – 26.667	0.320
Respiratory Failure	0.348	0.029 – 3.111	0.363
Vascular Failure	1.072	0.096 – 9.805	0.952
Sepsis	5.904	0.270 – 158.151	0.263

*Extreme outliers were defined independently for each predictor as values exceeding Q3 + 3 × IQR or falling below Q1 - 3 × IQR. Abbreviations: CI — Confidence Interval; OR — Odds Ratio; LAC — Lactate; CRP — C-reactive protein; PCT — Procalcitonin; ALB — Albumin; LAR — Lactate-to-Albumin Ratio; CAR — C-Reactive Protein-to-Albumin Ratio; PAR — Procalcitonin-to-Albumin Ratio.

Panel C. Discriminatory performance of maximum and mean LAR, CAR, and PAR for ICU mortality after exclusion of extreme outliers (n = 113).

Marker	Cut-Off	AUC (95% CI)	Accuracy	Sensitivity	Specificity	PPV	NPV
**Maximum values**							
Maximum LAR	0.1031	0.656 (0.553 – 0.759)	0.673 (0.575 - 0.752)	0.458 (0.314 - 0.608)	0.831 (0.717 - 0.912)	0.667 (0.508 - 0.786)	0.675 (0.529 - 0.815)
Maximum CAR	3.3769	0.711 (0.615 – 0.808)	0.664 (0.611 - 0.779)	0.875 (0.748 - 0.953)	0.508 (0.381 - 0.634)	0.568 (0.439 - 0.791)	0.846 (0.699 - 0.902)
Maximum PAR	0.0117	0.622 (0.518 – 0.725)	0.566 (0.531 - 0.708)	0.938 (0.828 - 0.987)	0.292 (0.186 - 0.418)	0.495 (0.351 - 0.831)	0.864 (0.67 - 0.917)
**Mean values**							
Mean LAR	0.1064	0.667 (0.566 – 0.768)	0.681 (0.558 - 0.752)	0.333 (0.204 - 0.484)	0.938 (0.85 - 0.983)	0.8 (0.598 - 0.882)	0.656 (0.494 - 0.878)
Mean CAR	3.3581	0.728 (0.633 – 0.823)	0.699 (0.628 - 0.788)	0.812 (0.674 - 0.911)	0.615 (0.486 - 0.733)	0.609 (0.48 - 0.786)	0.816 (0.679 - 0.884)
Mean PAR	0.0286	0.642 (0.540 – 0.744)	0.619 (0.549 - 0.726)	0.771 (0.627 - 0.88)	0.508 (0.381 - 0.634)	0.536 (0.408 - 0.715)	0.75 (0.6 - 0.834)

*Cut-offs were derived using the Youden index. Extreme outliers were defined as values exceeding Q3 + 3 × IQR or falling below Q1 - 3 × IQR. Abbreviations: AUC — Area Under the Curve; PPV — Positive Predictive Value; NPV — Negative Predictive Value; LAR — Lactate-to-Albumin Ratio; CAR — C-Reactive Protein-to-Albumin Ratio; PAR — Procalcitonin-to-Albumin Ratio.
